# Autophagy and Skeletal Muscles in Sepsis

**DOI:** 10.1371/journal.pone.0047265

**Published:** 2012-10-09

**Authors:** Mahroo Mofarrahi, Ioanna Sigala, Yeting Guo, Richard Godin, Elaine C. Davis, Basil Petrof, Marco Sandri, Yan Burelle, Sabah N. A. Hussain

**Affiliations:** 1 Department of Critical Care Medicine, McGill University Health Centre, and Meakins-Christie Laboratories, McGill University, Montréal, Québec, Canada; 2 George P. Livanos Laboratory, Department of Critical Care and Pulmonary Services, University of Athens Medical School, Evangelismos Hospital, Athens, Greece; 3 Faculty of Pharmacy, Université de Montréal, Montréal, Québec, Canada; 4 Department of Anatomy and Cell Biology, McGill University, Montréal, Québec, Canada; 5 Dulbecco Telethon Institute, Venetian Institute of Molecular Medicine, Padova, Italy; University of Pittsburgh, United States of America

## Abstract

**Background:**

Mitochondrial injury develops in skeletal muscles during the course of severe sepsis. Autophagy is a protein and organelle recycling pathway which functions to degrade or recycle unnecessary, redundant, or inefficient cellular components. No information is available regarding the degree of sepsis-induced mitochondrial injury and autophagy in the ventilatory and locomotor muscles. This study tests the hypotheses that the locomotor muscles are more prone to sepsis-induced mitochondrial injury, depressed biogenesis and autophagy induction compared with the ventilatory muscles.

**Methodology/Principal Findings:**

Adult male C57/Bl6 mice were injected with i.p. phosphate buffered saline (PBS) or *E. coli* lipopolysaccharide (LPS, 20 mg/kg) and sacrificed 24 h later. The tibialis anterior (TA), soleus (SOLD) and diaphragm (DIA) muscles were quickly excised and examined for mitochondrial morphological injury, Ca^++^ retention capacity and biogenesis. Autophagy was detected with electron microscopy, lipidation of Lc3b proteins and by measuring gene expression of several autophagy-related genes. Electron microscopy revealed ultrastructural injuries in the mitochondria of each muscle, however, injuries were more severe in the TA and SOL muscles than they were in the DIA. Gene expressions of nuclear and mitochondrial DNA transcription factors and co-activators (indicators of biogenesis) were significantly depressed in all treated muscles, although to a greater extent in the TA and SOL muscles. Significant autophagosome formation, Lc3b protein lipidation and upregulation of autophagy-related proteins were detected to a greater extent in the TA and SOL muscles and less so in the DIA. Lipidation of Lc3b and the degree of induction of autophagy-related proteins were significantly blunted in mice expressing a muscle-specific IκBα superrepresor.

**Conclusion/Significance:**

We conclude that locomotor muscles are more prone to sepsis-induced mitochondrial injury, decreased biogenesis and increased autophagy compared with the ventilatory muscles and that autophagy in skeletal muscles during sepsis is regulated in part through the NFκB transcription factor.

## Introduction

Severe sepsis elicits mitochondrial injury, dysfunction and depressed biogenesis in skeletal muscles. During the initial phase of sepsis, these changes are manifested as rapid increases in mitochondrial ATP production [1]. As a consequence of the augmented oxidative phosphorylation that occurs during this phase, significant increases in oxygen and nitrogen radical production take place, which, in turn, trigger extensive mitochondrial injury in skeletal and cardiac muscles [2], [3]. The initial phase of sepsis is usually followed by a second phase, where numbers of mitochondria and activities of various mitochondrial enzymes in skeletal muscles are significantly reduced, rendering cellular ATP production more dependent on glycolysis [4]–[8]. The second phase has been described by Singer [1] as a pro-survival state of cellular hibernation, wherein protein recycling programs, such as the autophagy-lysosomal degradation pathway, must be activated to provide alternate energy supplies. Mitochondrial biogenesis is re-activated during the late phase of sepsis if the subject or animal survives earlier phases.

The permeability transition pore (PTP) is a Ca^2+^-dependent channel located in the mitochondrial inner membrane. Pathological opening of the PTP is well known to trigger reactive oxygen species (ROS) production, decreased ATP synthesis, induction of apoptotic and necrotic cell death. Pathological PTP opening has been shown to contribute to cardiac and lung dysfunction in sepsis [9] and to initiate mitochondrial swelling and drastic changes in inner membrane morphology [10] as well as to play a role in initiating the process of autophagy [11], [12]. It is not known, however, whether or not sepsis elicits significant changes in susceptibility to PTP opening of skeletal muscle mitochondria.

Autophagy is a highly-conserved adaptive response designed to recycle unnecessary, redundant, or inefficient cellular components. Cytosolic proteins and organelles such as mitochondria are sequestered in double membrane vesicles called autophagosomes and delivered to lysosomes for degradation and subsequent recycling [13]. Recent studies indicate that autophagy is particularly active in skeletal muscles under basal condition and in response to atrophic stimuli such as starvation, denervation and oxidative stress [14]–[16]. The kinetics of autophagy is different in skeletal muscles under stress as compared to other tissues. Most tissues undergo transient induction of autophagy in response to stress stimuli and the process only lasts for a few hours. In contrast, persistent generation of autophagosomes continues for days in skeletal muscles [17]. Recent studies have indicated that mitochondrial dysfunction and increased reactive oxygen species within the mitochondria trigger both induction of autophagy and selective targeting of damaged mitochondria by autophagosomes [18]. Moreover, genetic inactivation of autophagy by selective deletion of Atg7 has recently been shown to induce a severe myopathic phenotype of skeletal muscle characterized by accumulation of abnormal mitochondria [19]. Taken together, these observations suggest that autophagy is an essential process in skeletal muscles, designed to maintain growth, development, and quality control of mitochondrial networks [13]. Very little is known about the extent, time course, and the regulation of autophagy in skeletal muscles in sepsis. A recent study by Doyle *et al.* [20] described that activation of Toll-like receptor 4, an important mediator of organ dysfunction in sepsis, triggers significant increase in autophagy in cultured skeletal muscle cells and that this induction is mediated by p38 mitogen activated protein kinase pathway. Whether in vivo sepsis triggers significant increase in autophagy in skeletal muscles and whether the extent of autophagy in septic skeletal muscles is related to degree of mitochondrial dysfunction remain unclear.

In this study, we used a murine model of lipopolysaccharide (LPS)-induced sepsis which simulates the extreme forms of human sepsis, like severe sepsis and septic shock, to test three hypotheses regarding mitochondrial function and autophagy in skeletal muscles undergoing sepsis. The first is that sepsis triggers substantial mitochondrial morphological injury, inhibition of mitochondrial biogenesis and increased opening of mitochondrial permeability transition pores and that the extent of mitochondrial injury is more severe in locomotor muscles compared with ventilatory muscles. The second hypothesis is that skeletal muscle mitochondrial dysfunction in septic skeletal is associated with significant increase in autophagy and that the induction of autophagy is more severe in locomotor muscles compared with the ventilatory muscles. The rationale for proposing that mitochondrial dysfunction and autophagy induction are more severe in locomotor muscles vs. ventilatory muscles rests on clinical and experimental evidence suggesting that the ventilatory muscles which undergo phasic activation and stretching during the breathing cycle, is relatively, spared from sepsis-induced mitochondrial dysfunction [21]. The third hypothesis to be tested in this study is that the induction of autophagy in septic skeletal muscles is mediated in part through NFκB transcription factor. This hypothesis is based on published evidence implicating NFκB in the regulation of autophagy in other cells [22], [23].

**Table 1:** Primers used for real-time PCR experiments to detect the expression of various genes in the TA, SOL and DIA muscles of PBS- and LPS-injected mice.

Gene	Position		Accession#
Nrf1	Forward	5′- CGCTCATCCAGGTTGGTACA-3′	NM_010938
	Reverse	5′- TCCATCAGCCACAGCAGAGT-3′	
Nrf2	Forward	5′- GATGCCTGCAATGTGAGAGC-3′	NM_008065
	Reverse	5′- AAGCAGCGGAGAGGAAACAG-3′	
Pgc1α	Forward	5′- AATCAGACCTGACACAACGC-3′	NM_010938
	Reverse	5′- GCATTCCTCAATTTCACCAA-3′	
Pgc1β	Forward	5′- GACCCCTTCAAGCCAGACAC-3′	NM_010938
	Reverse	5′- TGAGACTGGTTGGGTTGTGG-3′	
CoxIVi1	Forward	5′- TGGGAGTGT TGTGAAGAGTGA-3′	NM_009941.2
	Reverse	5′- GCAGTGAAGCCGATG AAGAAC-3′	
Tfam	Forward	5′-GCACCCTGCAGAGTGTTCAA-3′	NM_009360
	Reverse	5′-CGCCCAGGCCTCTACCTT-3′-3′	
Tfb1m	Forward	5′- GGAAGCAAACAGCACAGTCG-3′	NM_146074
	Reverse	5′- GCTGCTTGATCTTGGGCTCT-3′	
Tfb2m	Forward	5′- AAGGACTGGCAAACGAGGAA-3′	NM_008249
	Reverse	5′- TCCTGGCCGCTTTCTTACAT-3′	
CoxI	Forward	5′-GAAGAGACAGTGTTTCATGTGGTGT-3′	AK_159675
	Reverse	5′- TCCTGGCCGCTTTCTTACAT-3′	
Mapl1 (Lc3b)	Forward	5′-CGATACAAGGGGGAGAAGCA-3′	NM_026160
	Reverse	5′- ACTTCGGAGATGGGAGTGGA-3′	
Beclin1	Forward	5′- TGAATGAGGATGACAGTGAGCA-3′	NM_019584
	Reverse	5′- CACCTGGTCTCCACACTCTTG-3′	
Uvrag	Forward	5′-CCCTGTGAACACAAGGGTCA-3′	NM_178635
	Reverse	5′- CCCAGCGCTTTCTTCTGTCT-3′	
Atg14L	Forward	5′- TGCAACCACTGCACACACTC-3′	NM_172599
	Reverse	5′- CCTCGAGGTCTGCTCGAACT-3′	
Atg12	Forward	5′- TCCGTGCCATCACATACACA-3′	NM_026217
	Reverse	5′- TAAGACTGCTGTGGGGCTGA-3′	
Gabarapl1	Forward	5′- CATCGTGAGAAGGCTCCTA-3′	NM_020590
	Reverse	5′- ATACAGCTGGCCCATGGTAG-3′	
Pi3kc3	Forward	5′- TGTCAGATGAGGAGGCTGTG-3′	NM_181447
	Reverse	5′- CCAGGCACGACGTAACTTCT-3′	
Atg4b	Forward	5′- ATTGCTGTGGGGTTTTTCTG-3′	NM_174574
	Reverse	5′- AACCCAGGTTTTCAGAGG-3′	
Lamp2a	Forward	5′- TGGCTAATGGCTCAGCTTTC-3′	NM_0010179
	Reverse	5′- ATGGGCACAAGGAGTTGTC-3′	
Bcl-XL	Forward	5′- GTGAAGCAAGCGCTGAGAGA-3′	NM_009743
	Reverse	5′- ACGATGCGACCCCAGTTTAC-3′	
Bad	Forward	5′- TGGGGAGCAACATTCATCAG-3′	NM_007522
	Reverse	5′- AGCTCCTCCTCCATCCCTTC-3′	
Bim	Forward	5′- GCAATGGCTTCCATACGACA-3′	NM_207680
	Reverse	5′- TTGCAAACACCCTCCTTGTG-3′	
Bax	Forward	5′- GGAGATGAACTGGATAGCAATATGG-3′	NM_207680
	Reverse	5′- GTTTGCTAGCAAAGTAGAAGAGGGC-3′	
Bnip3	Forward	5′-TTCCACTAGCACCTTCTGATGA-3′	NM_009760
	Reverse	5′-GAACACCGCATTTACAGAACAA-3′	
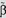 -Actin	Forward	5′- CTGGCTCCTAGCACCATGAAGAT-3′	NM_007393
	Reverse	5′- GGTGGACAGTGAGGCCAGGAT-3′	

## Methods

### Animal Preparation

All procedures were approved by the Animal Ethics Committees of McGill University (protocol# 5749) and the Université de Montréal and were in accordance with the guidelines of the Canadian Council of Animal Care. Adult (8- to 12-wk-old) male wild-type C57/BL6j mice were injected i.p. with a single dose of phosphate-buffered saline (PBS) (control) or E. coli lipopolysaccharide (LPS) (20 mg/kg serotype 055:B5; Sigma-Aldrich, Oakville, ON). Higher doses of LPS result in relatively high mortality. Animals were euthanized with sodium pentobarbital after 24 h and rapid excision of TA, SOL and DIA muscles followed. Each muscle was weighed. Some muscle samples were immediately used to study mitochondrial function in permeabilized fibers while some were flash-frozen in liquid nitrogen and stored at −80°C for later use. To evaluate the role of NFκB in the regulation of autophagy, we used transgenic mice expressing a muscle-specific IκBα superrepresor (MISR) in which serine 32 and 36 residues of IκBα are mutated to alanine [24]. MISR is immune from phosphorylation by IKKs and thereby renders NFκB in sensitive to IKK-induced stimuli selectively in skeletal muscles {3816}. Adult (8- to 12-wk-old) male MISR mice and their wild type (WT) littermates were injected with PBS or LPS. After 24 h, the animals were euthanized and the TA muscle was collected and examined for autophagy indices. We focused on the TA in these mice because MISR expression is relatively higher in muscles rich in fast twitch muscle fibers such as the TA compared with the DIA and SOL [24].

**Figure 1:**
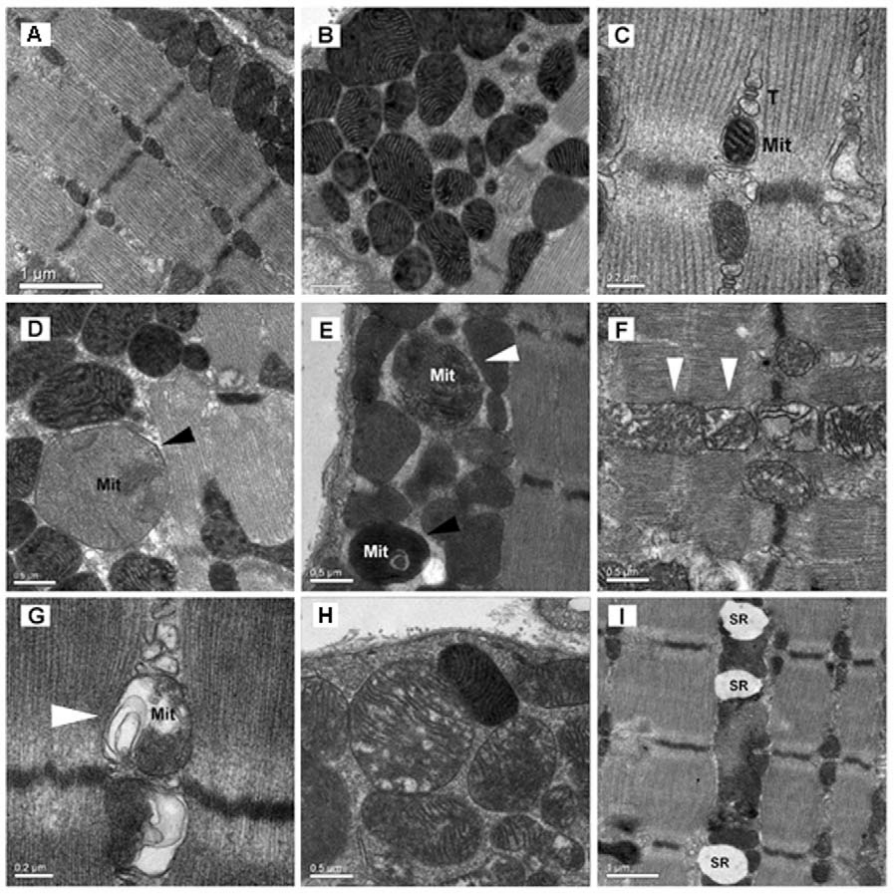
Representative electron microscopy images (A,B,C) of normal sub-sarcolemmal and intermyofibrillar mitochondria in SOL, DIA and TA muscles, respectively, of control mice. Mit indicates mitochondria. T indicates triad. Representative electron microscopy images (D,E,F,) of mitochondrial morphological abnormalities in SOL, DIA and TA muscles of LPS-treated mice. Swollen mitochondrion (D, black arrow), mitochondrion densely packed with protein aggregates (E, black arrow), mitochondria with extensive loss of internal cristae (F, white arrows). Representative electron microscopy images of mitochondria with myelin-like structures (G, white arrow), extensive vacuole formation (H) and dilated sarcoplasmic reticula (I) in TA muscles of LPS-treated mice.

**Figure 2:**
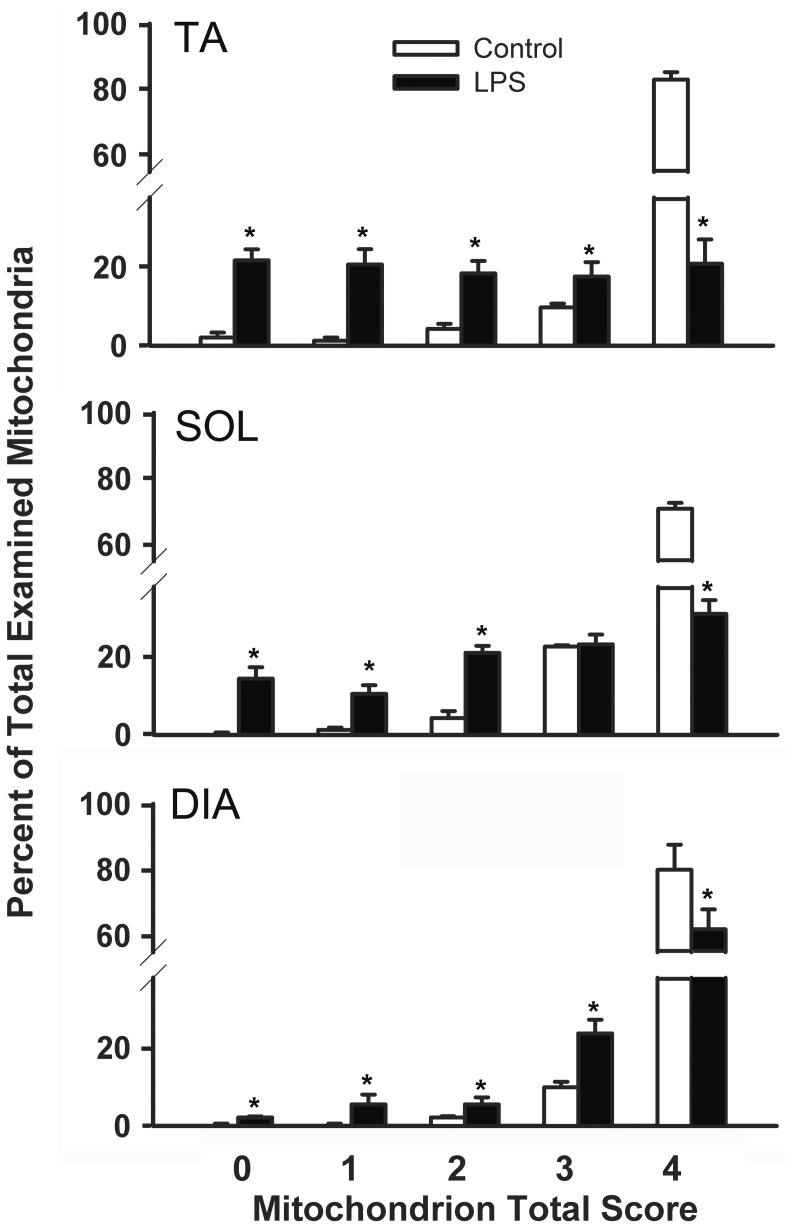
Means ± SEM of proportion of mitochondria with ultrastructure morphology scores between 0 to 4 as percent of total counted mitochondria in TA, SOL and DIA of control and LPS-treated mice. Mitochondria were assigned a score ranging from 0 to 4 based on integrity of matrix, internal cristae and membrane and EM density. N = 5 per group. *P<0.05, as compared to control group.

### Electron Microscopy

Adult (8- to 12-wk-old) male wild-type C57/BL6j mice were injected i.p. with PBS (control group, n = 5) or *E. coli* LPS (LPS group, n = 5), euthanized 24 h later with sodium pentobarbital and perfused at a pressure in the physiological range through the left ventricle with normal saline followed by 3% glutaraldehyde buffered with 0.1 M sodium cacodylate (pH 7.4). Following perfusion, TA, SOL and DIA muscles were removed, dissected into segments and placed in fresh fixative at room temperature for an additional 2 h. Following fixation, tissues were washed overnight in cacodylate buffer at 4°C then sequentially treated with 1% OsO4 in buffer, 2% tannic acid in buffer, and 2% uranyl acetate in distilled water. Tissues were then dehydrated via a graded series of methanol to propylene oxide, infiltrated and embedded in EPON™, as previously described [25]. Thin sections (60 nm) were counterstained with methanolic uranyl acetate and lead citrate and viewed using a Tecnai 12 transmission electron microscope at 120 kV. Images were digitally captured. A total of 400 mitochondria per muscle sample were visualized. Each mitochondrion was assessed for integrity of matrix, cristae and outer membrane and EM density. Each of these four parameters was scored from 1 (normal) to zero (damaged). A mitochondrion with intact matrix, well defined cristae, intact outer membrane and homogenous EM density was given a maximum score of 4 while one with disrupted matrix, disorganized cristae, damaged outer membrane and low EM density was given a total score of zero. Both subsarcolemmal (SL) and intermyofibrillar (IM) mitochondria were scored.

**Figure 3:**
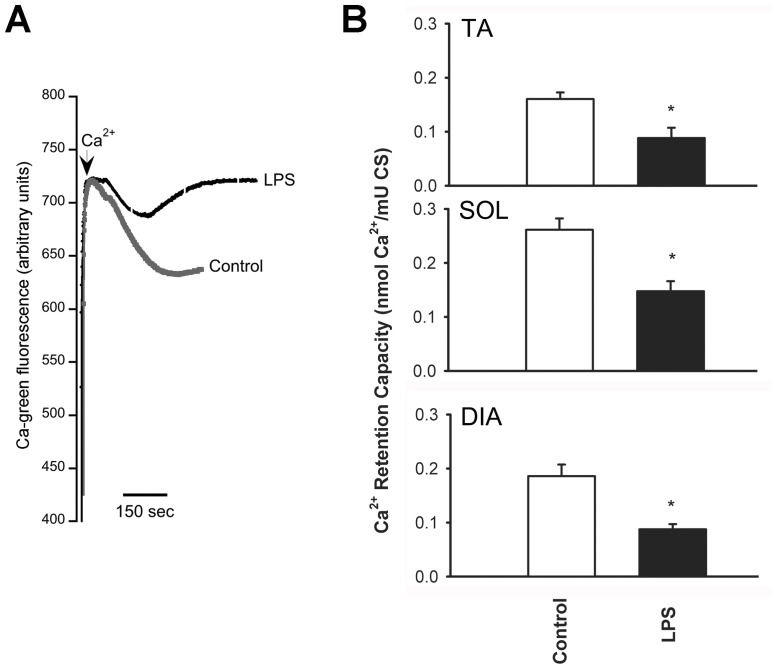
Representative example (A) and means ± SEM of mitochondrial Ca^2+^ retention capacity of the TA, SOL and DIA muscles of control and LPS-treated mice (B). *P<0.05, as compared to control group. N = 6 per group.

**Figure 4:**
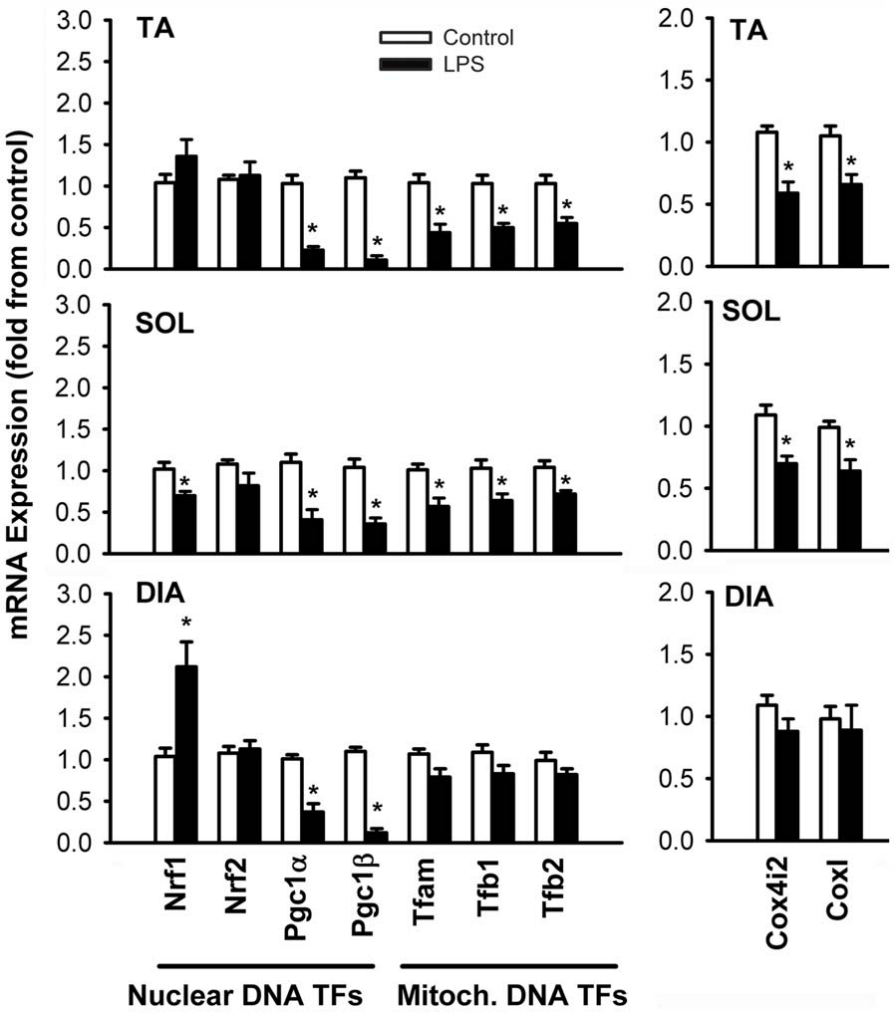
Means ± SE of mRNA expressions of transcription factor regulators of nuclear-encoded mitochondrial proteins (Nrf1, Nrf2, Pgc1α, Pgc1β), regulators of mitochondrial DNA transcription (Tfam, Tfb1 and Tfb2) and cytochrome c oxidase subunits Cox4i2 and Cox1 in the TA, SOL and DIA muscles of LPS-treated mice. N = 6 per group. Values expressed as fold change relative to control group. *P<0.05, as compared to control group.

### Sensitivity to Ca^2+^-induced Permeability Transition Pore (PTP) Opening

Dissection and permeabilization of fiber bundles with saponin were performed on the TA, SOL and DIA as previously described [26]. Ghost fibers were prepared by incubating saponin permeabilized muscle bundles in a high KCl medium to extract myosin. Following extensive rinsing, ghost fibers were incubated with continuous stirring at 23°C in quartz microcuvettes containing CRC buffer (in mM: 250 sucrose, 10 MOPS, 0.005 EGTA, 10 P_i_-TRIS, pH 7.3) supplemented with glutamate-malate (5∶2.5 mM) and 0.5 nM oligomycinate. Following addition of fiber bundles and respiratory substrates, mitochondria were exposed to a single pulse of 20 nM Ca^2+^. Changes in extra-mitochondrial Ca^2+^ concentrations were monitored fluorometrically using Calcium-green 5N. PTP opening susceptibility was assessed by measuring time required for PTP opening and Ca^2+^ retention capacity (CRC) [26]. CRC was taken as the total amount of Ca^2+^ accumulated by mitochondria before release by PTP opening. CRC values are expressed in nmoles of Ca^2+^ per mg of dry weight. A standard curve relating [Ca^2+^] to the fluorescence of Ca-green was performed to calculate the [Ca^2+^] in the microcuvette. All measurements were performed in duplicate for each sample.

**Figure 5:**
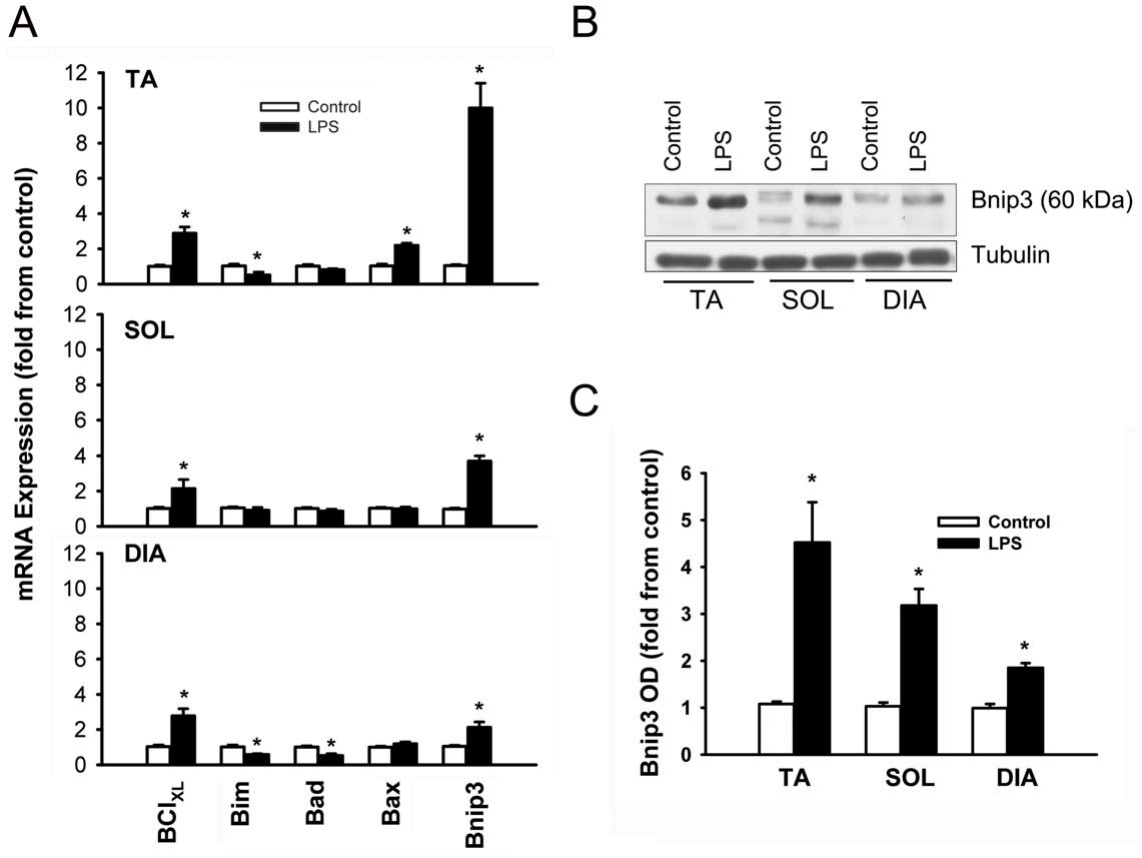
Means ± SEM of mRNA expressions of Bcl_XL_ (anti-apoptotic) and Bim, Bad, Bax and Bnip3 (pro-apoptotic) genes in the TA, SOL and DIA muscles of LPS-treated mice (A). Representative immunoblots of Bnip3 protein in the TA, SOL and DIA muscles of control and LPS-treated mice (B). Means ± SEM of Bnip3 protein (60 kDa)(C). N = 6 per group. Values expressed as fold change relative to control group. *P<0.05, as compared to control group.

### RNA Extraction and Real-time PCR

Total RNA was extracted from the TA, SOL and DIA muscles using a GenElute™ Mammalian Total RNA Miniprep Kit (Sigma-Aldrich, Oakville, ON). Quantification and purity of total RNA was assessed by A260/A280 absorption. Total RNA (2 µg) was then reverse transcribed using a Superscript II® Reverse Transcriptase Kit and random primers (Invitrogen Canada, Inc., Burlington, ON). Reactions were incubated at 42°C for 50 min and at 90°C for 5 min. Real-time PCR detection of mRNA expression was performed using a Prism® 7000 Sequence Detection System (Applied Biosystems, Foster City, CA). mRNA expressions of the following groups of genes were quantified using specific sets of primers, as listed in Table 1.

#### A) Electron transport chain proteins and regulators of mitochondrial gene expression

We assessed mRNA levels of the transcription factors nuclear respiratory factor 1 and 2α (Nrf1 and Nrf2α), the peroxisome proliferator-activated receptors co-activator Pgc1α and Pgc1β, transcription factor A mitochondrial (Tfam), transcription factor B1 mitochondrial (Tfb1), transcription factor B2 mitochondrial (Tfb2) and cytochrome c oxidase subunits I (CoxI) and IV isoform 1 (Cox4i1).

#### B) Mitochondrial regulators of apoptosis

mRNA levels for the anti-apoptotic protein Bcl_XL_, and the pro-apoptotic proteins Bim, Bad, Bax, and Bnip3 were also detected.

#### C) Autophagy-related genes

Several autophagy-related gene expressions were assessed due to their importance to: a) the initial phase of autophagosome formation (Beclin1, Vps34/Pi3kc3, Uvrag, and Atg14L); b) the expansion of the isolation membrane (Lc3b, Gabararapl1 and Atg4B); c) vesicle elongation (Lc3b and Atg12); and d) chaperon-mediated autophagy (Lamp2a).

**Figure 6:**
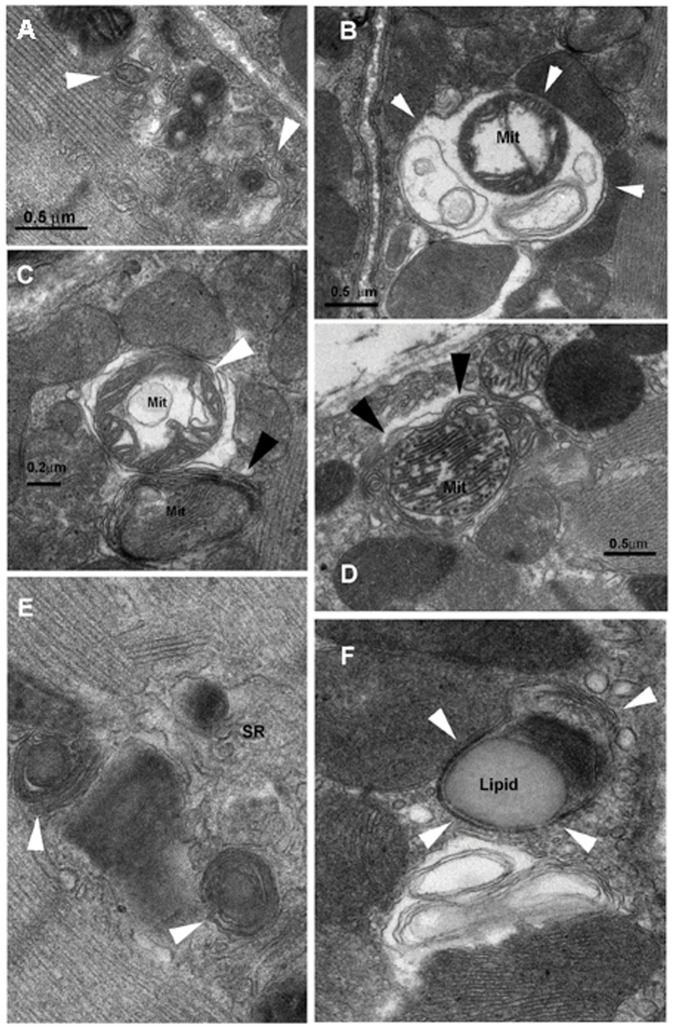
Representative electron microscopy images of autophagosome formation in skeletal muscles of LPS-treated mice. Double-membrane autophagosomes in close proximity containing one (A) or more than one mitochondrion (B) in TA muscle. Double-membrane autophagosomes containing damaged mitochondria in the SOL (C, white arrow) and DIA (D, black arrows). Four-membrane autophagosome (C, black arrow) is also shown. Autophagosomes containing intermyofibrillar mitochondria and lipid droplets in TA (E and F, respectively) were also evident.

**Figure 7:**
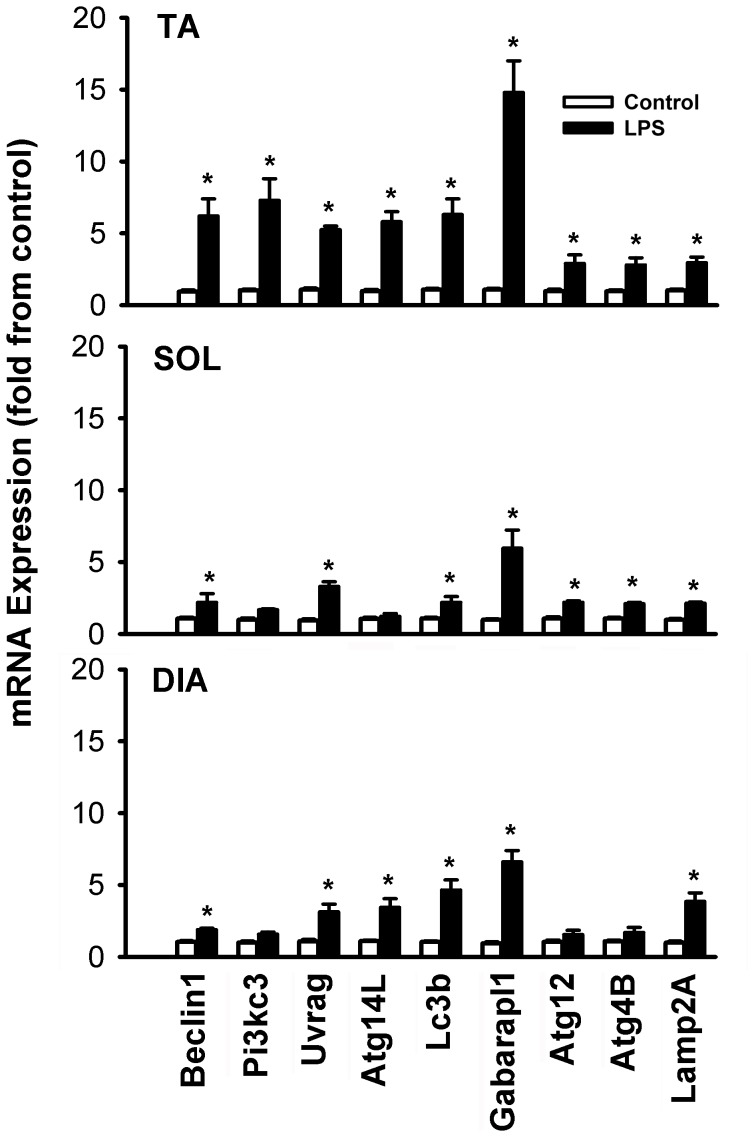
Means ± SEM of mRNA expressions of autophagy-related genes in the TA, SOL and DIA muscles of LPS-treated mice. N = 6 per group. Values expressed as fold change relative to control group. *P<0.05, as compared to control group.

**Figure 8:**
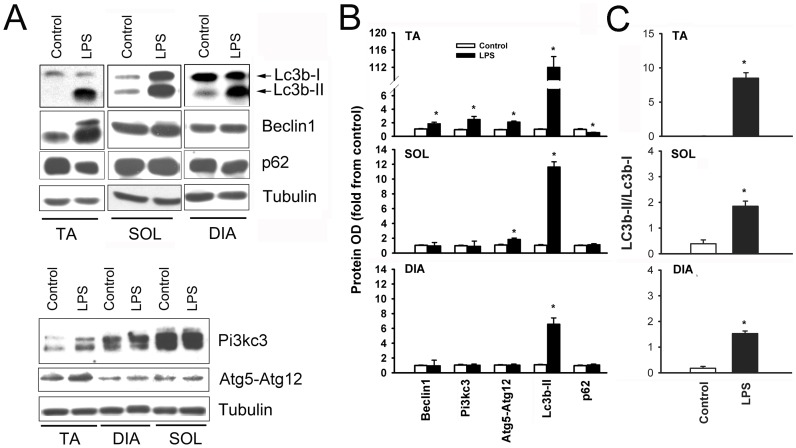
Representative immunoblots of Lc3b, Beclin1, p62 and tubulin (A) and Pi3kC3 and Atg5-Atg12 complex in the TA, SOL and DIA muscles of control and LPS-treated mice. Means ± SEM of autophagy-related protein optical densities (B) and the ratio of Lc3b-II/Lc3b-I protein optical densities (C) in the TA, SOL and DIA muscles of LPS-treated mice. N = 6 per group. Values expressed as percent relative to control group. *P<0.05, as compared to control group.

In all assays, 1.0 µl of reverse-transcriptase reagent was added to 25 µl of SYBR® Green master mix (Qiagen Inc, Valencia, CA) and 3.5 µl of 10 µM primer. The thermal profile used was as follows: 95°C for 10 min; 40 cycles each of 95°C for 15 s; 57°C for 30 s; and 72°C for 33 s. All real-time PCR experiments were performed in triplicate. A melt analysis for each PCR experiment was performed to assess primer-dimer formation or contamination. Relative mRNA level quantifications of target genes were determined using the threshold cycle (ΔΔCT) method using the housekeeping gene β-actin.

**Figure 9:**
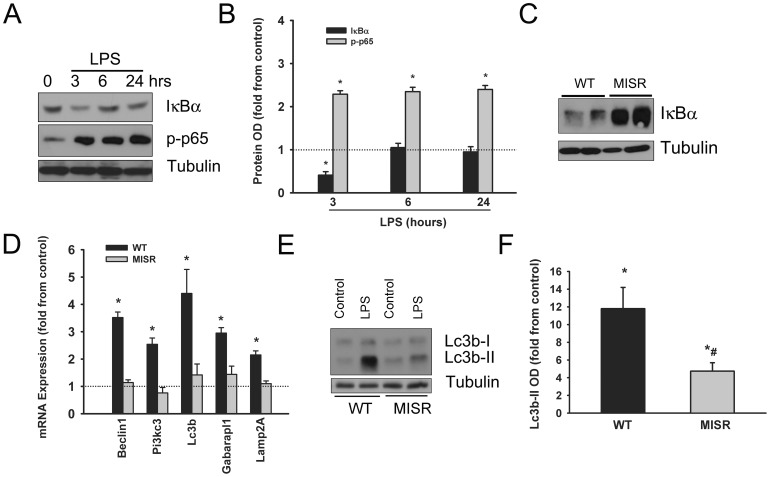
Representative immunoblots of IκBα, phospho-p65 (RelA) and tubulin proteins (A) and means ± SEM of optical densities of IκBα and phospho-p65 (n = 6, B) in the TA muscles of WT mice after 3, 6 and 24 h of LPS injection. Representative immunoblots of IκBα and tubulin proteins in the TA muscles of WT and MISR mice (C). Means ± SEM of mRNA levels of autophagy-related genes in the TA muscle of WT and MISR mice measured after 24 h of LPS injection and normalized as fold change from control (n = 6, D). Representative immunoblots (E) and means ± SEM of Lc3b-II optical density (n = 6, F) of TA muscle measured after 24 h of LPS injection. Data are shown as fold changes from control values. *P<0.05, as compared to control group. #P<0.05 compared with wild type mice.

### Protein Extraction and Immunoblotting

Protein expressions of several genes were quantified using immunoblotting with selective antibodies, including Bnip3, Lc3b, Beclin1, p62 (Sqstm1), Pi3kc3, Atg12, phospoh-p65 subunit of NFκB (Ser^536^), IκBα and β-tubulin. All antibodies except β-tubulin (Sigma) were obtained from Cell Signaling Inc. Frozen muscle samples were homogenized in homogenization buffer (10 mM tris-maleate, 3 mM EGTA, 275 mM sucrose, 0.1 mM DTT, 2 µg/ml leupeptin, 100 µg/ml PMSF, 2 µg/ml aprotinin, and 1 mg/100 ml pepstatin A, pH 7.2). Samples were centrifuged at 1,000 g for 10 min. Pellets were discarded and supernatants were designated as crude homogenate. Total muscle protein levels in each sample were determined using the Bradford protein assay technique. Crude homogenate samples (50 µg/sample) were mixed with SDS sample buffer, boiled for 5 min at 95°C, then loaded onto tris-glycine sodium dodecylsulfate polyacrylamide gels (SDS-PAGE) and separated by electrophoresis. Proteins were transferred by electrophoresis to polyvinylidene difluoride (PVDF) membranes and blocked with 1% bovine serum albumin or milk for 1 h at room temperature. PVDF membranes were incubated overnight with primary antibodies at 4°C. PVDF membranes were then washed and incubated with horseradish peroxidase-conjugated secondary antibody. Specific proteins were detected with an enhanced chemiluminescence kit (Millipore, Billerica, MA). Equal loading of proteins was confirmed by stripping membranes and re-probing with anti-tubulin antibody. Blots were scanned with an imaging densitometer and optical densities (OD) of protein bands were quantified using ImagePro® Plus software (Media Cybernetics, Carlsbad, CA).

### Statistical Analysis

Results are expressed as means±SEM. Statistical differences were analyzed using two-way ANOVA for multiple group and time comparisons (Statistica Software, StatSoft, Inc., Tulsa, OK). Tukey post hoc tests were used to identify significant differences (P<0.05).

## Results

### Changes in Muscle Weight

The weight of TA, SOL and DIA muscles in the control group averaged 0.00367±0.0000375, 0.000637±0.0000109 and 0.003254±0.0000626 g/g body weight, respectively (n = 6). LPS injection elicited a significant (p<0.05) decline in the weight of TA (0.00297±0.0000309 g/g body weight), SOL (0.000598±0.0000122 g/g body weight) and DIA (0.002473±0.0000722 g/g body weight) muscles, as compared with the control group.

### Mitochondrial Ultrastructure

Figure 1 shows normal sarcolemmal and intermyofibrillar mitochondria in the TA, SOL and DIA muscles of control mice. In animals that received LPS, major morphological abnormalities were detected in both types of mitochondria. Many mitochondria were abnormally large in size (black arrow in Figure 1D), presented abnormal and/or missing internal cristae (white arrows in Figure 1E and F), had tightly packed matrices with electron dense protein aggregates (black arrow in Figure 1E) and those showing large translucent vacuoles (Figure 1H). Major morphological abnormalities were observed in a group of adjacent mitochondria in one muscle fiber (Figure 1F) or in a single mitochondrion. Many mitochondria presented with myelin-like structures, indicating dysfunctional regulation of inner membrane morphology (Figure 1G). In addition to mitochondrial injury, significantly dilated SR structures were detected in the DIA and limb muscles of LPS-injected animals (Figure 1I). Quantitative analysis of mitochondrial ultrastructure dysfunctions revealed that ∼80% of mitochondria in the three muscles of control animals had normal structure and morphology (a total score of 4) (Figure 2). LPS administration increased the proportion of mitochondria with morphological abnormalities in all muscles, although the degree of mitochondrial ultrastructure damage was greater in TA muscles (>20% of mitochondria had a score of 0) and SOL, as compared to the DIA (<5% of mitochondria had a score of 0) (Figure 2).

### Sensitivity to Ca^2+^-induced PTP Opening

Mitochondrial Ca^2+^ retention capacity was significantly reduced in all three muscles, indicating an increased susceptibility to PTP opening (Figure 3).

### Mitochondrial Biogenesis Gene Expression

Among the transcriptional regulators of nuclear-encoded mitochondrial proteins, mRNA expressions of the co-activators Pgc1α and Pgc1β showed significant declines in all three muscles, although declines were relatively largest in the TA muscle (Figure 4). Nrf2 expression remained unchanged while Nrf1 expression rose in the DIA and declined in the SOL muscle (Figure 4). Expression levels of Tfam, Tfb1, Tfb2 and Cox subunits I and IV significantly declined in the TA and SOL muscles, while remaining unchanged in the DIA (Figure 4).

### Mitochondrial Regulation of Apoptosis

Significant increases were seen in mRNA expressions of the anti-apoptotic protein Bcl_XL_ in all three muscles (Figure 5). Expressions of pro-apoptotic genes, however, were reversed in the DIA and the TA muscles. For instance, Bim and Bad expressions declined in the DIA, reflecting an anti-apoptotic response, while Bim and Bax expressions increased significantly in the TA muscle (Figure 5). Bnip3 expression was significantly induced in all muscles but to a lesser extent in the DIA compared with TA and SOL (Figure 5). Immunoblotting experiments confirmed the induction of Bnip3 protein (60kDa) in all three muscles. Again, the relative level of induction in the DIA was less than that of the TA and SOL (Figure 5).

### Autophagy

Autophagic vacuoles containing structurally abnormal sub-sarcolemmal and intermyofibrillar mitochondria were clearly observed in all three muscles of the LPS group (Figure 6). Of note, while autophagosomes were sometimes observed in the muscles of the control group, their occurrence was rare. Relatively large autophagosomes containing more than one mitochondrion were also detected as were autophagosomes containing lipid droplets (Figure 6). To further quantify the degree of autophagy, real-time PCR and immunoblotting were performed for several genes involved in autophagosome formation. Figure 7 shows that LPS administration triggered significant increases in the mRNA expressions of Beclin1, Pi3kc3, Uvrag, Atg14L, Lc3b, Gabarapl1, Atg4b, Atg12, and Lamp2a. Inductions were relatively greater in the TA muscle as compared to the SOL and DIA muscles. Immunoblotting confirmed the induction of Beclin1, Pi3kc3 and Atg12 in the TA and SOL but there was no change in the expression of these proteins in the DIA (Figure 8). We also observed a significant decline in the level of p62 (Sqstm1, a known cargo of autophagosomes) in the TA muscle (Figure 8). Significant increases in the expressions of Lc3b-II (lipidated form of Lc3b) were also observed in the TA and SOL but to a lesser extent in the DIA (Figure 8).

We evaluated the time course of NFκB transcription activation in skeletal muscles in response to LPS injection by measuring total IκBα protein level and the level of p65 (RelA) phosphorylation on Ser^536^. Total IκBα level declined significantly while phosphorylation of p65 rose significantly after 3 hrs of LPS injection (Figure 9). Figure 9C confirms that the levels of IκBα was significantly greater in the TA muscle of MISR mice compared with WT mice. Under control condition, no differences were observed in Lc3b-II levels and mRNA expression of autophagy-related genes among the TA muscles of WT and MISR mice (results are not shown). LPS administration triggered significant elevation of Beclin1, Pi3kc3, Lc3b, Gabarapl1 and Lamp2A mRNA levels in WT mice but not in MISR mice (Figure 9D). Similarly, LPS injection in WT mice triggered a significant rise in Lc3b-II levels suggesting increased autophagosome formation whereas relatively lower levels of Lc3b-II induction were triggered by LPS injection in MISR mice compared with WT mice (Figure 9E and F). These results strongly suggest that NFκB activation plays an important role in LPS-induced autophagy in skeletal muscles.

## Discussion

The main findings of this study are: 1) LPS administration in mice elicits extensive mitochondrial morphological abnormalities in skeletal muscles that coincide with increased susceptibility to PTP opening and decreased mitochondrial biogenesis; 2) These changes in the muscle mitochondria were associated with significant induction of autophagy in skeletal muscles; 3) LPS-induced mitochondrial injury, inhibition of mitochondrial biogenesis, autophagosome formation and increased expressions of autophagy-related genes are more pronounced in the TA and SOL muscles as compared to the DIA.

This study provides novel morphological, functional and molecular evidence that autophagic removal of damaged mitochondria is activated in skeletal muscles undergoing severe sepsis and that NFκB transcription factor is involved in this activation. This autophagic effect occurs to a greater extent in locomotor muscles, which evince more pronounced mitochondrial ultrastructural abnormalities than those that are seen in the DIA. The present results also demonstrate that severe sepsis suppresses mitochondrial biogenesis signalling and that this effect is more pronounced in muscles with the greatest level of mitochondrial damage. Collectively, these data suggest that sepsis induces autophagic recycling of damaged mitochondria and that this recycling coincides with significant morphological and functional ultrastructural injury as well as depressed mitochondrial biogenesis.

### Impact of Sepsis on Mitochondrial Injury and Autophagy

The impact of sepsis on mitochondrial injury, both in human patients and in animal models, has been widely documented [5], [6], [27]–[29]. Many of these studies have used electron microscopy to assess morphological changes and measurements of respiratory function and oxidative enzyme activity/content to approximate energy production capacity In general, our descriptions of mitochondrial ultrastructure abnormalities in skeletal muscles of septic animals are consistent with previous reports in that they show mitochondria with extensive swelling, disrupted cristae, misfolding of the inner membrane and abnormal matrices [6], [28], [29]. However, in addition to morphological evidence, for the first time our study provides evidence that skeletal muscle mitochondria in septic animals are significantly more prone to opening of the PTP as compared with control animals. This effect may represent a direct contribution to muscle dysfunction, as pathological opening of the PTP is well known to trigger reactive oxygen species (ROS) production, decreased ATP synthesis, and induction of apoptotic and necrotic cell death [30], [31].

Several studies have shown that damaged and/or dysfunctional mitochondria are unable to maintain their membrane potential and are selectively targeted for autophagy [32], [33]. In skeletal muscles, increasing evidence suggests that autophagy is vital for the maintenance of proper muscle function [13]–[15]. However, few experimental data linking mitochondrial damage to induction of autophagy in muscle pathologies are as yet available. In the present study, we report that a general autophagic response is triggered in skeletal muscles, including the DIA, as indicated by the presence of autophagosomes engulfing mitochondria, upregulation of several autophagy-related genes, and by the increased lipidation of Lc3b protein. In addition, the expression of Bnip3, a BH3-only member of the Bcl-2 protein family that has a dual role in triggering apoptosis and autophagy [34], [35] and which plays a critical role in regulating starvation- and denervation-induced autophagy in skeletal muscles [36], [37] is significantly induced in skeletal muscles, both at the level of mRNA and protein. In contrast, mRNA expression levels of Bim, Bad and Bax, which are purely pro-apoptotic factors are either decreased or increased to a much lesser extent than is Bnip3, thus yielding an expression pattern of Bcl-2-related proteins that is more indicative of mitophagy than it is of Bcl-2-mediated mitochondrial apoptosis. Interestingly, we observed that induction levels of Bnip3 across muscles correlate with the extent of mitochondrial ultrastructure damage, with the greatest induction observed in the TA muscle and the least observed in the DIA. These results suggest that Bnip3 may indeed play an important role in the regulation of skeletal muscle mitophagy in sepsis. Although pathological opening of the PTP is well known to trigger cell death, loss of mitochondrial ΔΨ due to transient opening of the PTP has been shown to trigger mitophagy [12]. Therefore, our observation that mitochondria in the muscles of septic mice are more susceptible to PTP opening suggests that PTP opening itself contributes to the activation of autophagy. Taken as a whole, results from the present study provide strong evidence that autophagy is triggered in skeletal muscles of septic animals, that it is related to the levels of morphological and functional mitochondrial injury that are present, and that, therefore, autophagy is actively playing a role in removing mitochondria.

### Impact of Sepsis on Mitochondrial Biogenesis

Mitochondrial biogenesis is crucial for the replacement of damaged mitochondria. Results of this study suggest that severe sepsis is associated with impairment of mitochondrial biogenesis signalling. This is evident in all three muscles, albeit to different extents in respiratory and locomotor muscles. In the DIA, mRNA expression levels of Pgc1α and Pgc1β are significantly decreased in treated animals (Figure 4), which is consistent with results from a study where LPS-treatment of rat DIA resulted in decreased mRNA expressions of several nuclear encoded genes that are transcriptionally regulated by Pgc1 [5]. Sepsis, however, does not significantly alter the expressions of transcription factors that regulate mitochondrially-encoded genes such as Tfam, Tfb1, Tfb2 or CoxI. This may be due to a 2.2 fold increase in Nrf1 expression, which regulates the expressions of these factors [38]. In contrast, in locomotor muscles, Pgc1α and Pgc1β expressions are strongly downregulated, Nrf1expressions are unaffected, and significant decreases are evident in some (in the SOL) or all (in the TA) of the transcription factors that regulate mitochondrially-encoded genes, suggesting that mitochondrial biogenesis signalling is more significantly impaired in these muscles than in it is in the DIA. To our knowledge, only one study has investigated the impact of sepsis on mitochondrial biogenesis signalling in locomotor muscles. In that study, Fredriksson et al. reported that mRNA levels for human PGC1α, PGC1β and NRF1 were not significantly altered, while those of human TFAM, TFB1M and TFB2M were significantly increased in the *vastus lateralis* muscles of septic patients [27]. The reasons underlying the apparent discrepancy between the our results and theirs are not known, but could be related to species differences, duration and severity of sepsis or the type of skeletal muscle being investigated. Nevertheless, these results collectively indicate that sepsis leads to a perturbation of biogenesis signalling, which may impair the replacement of damaged organelles.

### Regulation of Autophagy by NFκB

The NFκB is a pro-inflammatory transcription factor that regulates the expression of a variety of genes including those involved in skeletal muscle proteolysis. Augmentation of NFκB activity has been described in different types of skeletal muscle atrophy [39]. The importance of NFκB in muscle wasting has been confirmed in experiments in which constitutive activation of NFκB caused severe muscle-wasting in mice [24]. Conversely, specific inhibition of NFκB in skeletal muscles by expression of IκBα superrepressor (MISR) has been shown to attenuate muscle atrophy in response to denervation, and unloading [24]. The influence of NFκB on autophagy is highly dependent on the cell type, cellular context and the triggers for NFκB stimulation. For instance, in sarcoma, breast and leukemia cancer cells, activation of NFκB by TNFα leads to inhibition of autophagy as a result of mTOR pathway activation [40], whereas NFκB activation during the recovery period from heat shock has been shown to activate autophagy and promote survival [22]. More recently, Criollo et al [23] reported that IκB kinase (IKK) complex activate autophagy in response to cellular starvation and this response is mediated through the AMPK and JNK1 pathways. In our study, we observed that LPS injection triggered significant increase in phosphorylation of p65 and degradation of IκBα confirming activation of NFκB in skeletal muscles. We evaluated the role of NFκB in regulating LPS-induced autophagy in skeletal muscles by comparing the regulation of autophagy-related genes and Lc3b lipidation in the TA of these mice to WT mice. Our results indicate that LPS injection failed to induce in the expression of several autophagy-related genes and was associated with much weaker increase in Lc3b protein lipidation in the TA of MISR mice compared with WT mice. These results clearly implicate NFκB activation is an important mediator of LPS-induced autophagy in skeletal muscles. One likely mechanism through which NFκB regulates autophagy in skeletal muscles is through direct regulation of autophagy-related gene expression. Previous studies have confirmed that the p65 (RelA) subunit of NFκB binds directly to selective binding sites on Beclin1 promoter and upregulates Beclin1, an important pathway through which NFκB induces autophagy [41]. It is possible that other autophagy-related genes including Lc3b, Pi3kc3, Gabarapl1 and Lamp2A may also be regulated directly by NFκB. We should emphasize that we don’t exclude the involvement of other transcription factors such as FoxO proteins in the regulation of autophagy in skeletal muscles in response to LPS administration.

### Fiber type Susceptibility to Mitochondrial Dysfunction and Autophagy

A major finding of this study is that LPS administration induces significantly greater severity of mitochondrial injury, autophagosome formation and upregulation of autophagy-related genes in the TA muscle, which is a locomotor muscle rich in glycolytic fast-twitch muscle fibers, as compared to the SOL, which is rich in oxidative slow-twitch fibers, or the DIA, which undergoes phasic patterns of contraction. The underlying reasons for this are, at present, unclear. One possibility is that glycolytic muscle fibers may contain higher levels of Toll-like 4 (TLR4) receptors, which are primarily responsible for initiating cellular responses to LPS. However, this is unlikely since Boyd *et al*. did not observe any differences in TLR4 expression in primary myoblasts and myotubes derived from mouse DIA and TA muscles [42]. Alternatively, the greater mitochondrial damage that was seen in the TA muscle as compared to the DIA may be due to differences in baseline mitochondrial content and/intrinsic mitochondrial properties that predispose muscles to or protect them from damage when they are challenged by systemic inflammation-induced sepsis. Indirect measurements of mitochondrial contents using citrate synthase (CS) activity assay (Supporting Information) revealed that the DIA has significantly higher CS activity (1369±63 µmol/min/mg protein) than SOL (1093±94 µmol/min/mg protein) and TA (852±42 µmol/min/mg protein). These results suggest that skeletal muscles with relatively low mitochondrial content (TA) are relatively more prone to develop mitochondrial injury and autophagy induction than those with relatively high mitochondrial content (DIA). The molecular mechanisms linking mitochondrial content to sepsis-induced mitochondrial injury and autophagy remain to be explored. In addition to mitochondrial content, differences in intrinsic mitochondrial properties may also explain why the TA develops more severe mitochondrial injury and induction of autophagy than the SOL and DIA. For example, we observed that net mitochondrial H_2_O_2_ release in the TA muscle was significantly higher than that observed in the DIA, possibly due to fiber-type differences in the rate of mitochondrial ROS generation, and/or its elimination by endogenous antioxidant systems [26], [43]. This was confirmed by measuring H_2_O_2_ generation in isolated DIA and TA muscle strips (Supporting Information). Such differences might predispose TA muscle mitochondria to more damage than DIA mitochondria, particularly during sepsis since sepsis is known to increase mitochondrial production of superoxide anions, nitric oxide and peroxynitrite [3].

Another factor that might explain differences in the degree of mitochondrial damage and autophagy is stimulation frequency. Slow-twitch muscle fibers, like those expressed in the DIA, are continuously being recruited and have higher stimulation frequencies than fast-twitch muscles like the TA [44]. The DIA is recruited intermittently at relatively high stimulation frequencies and is also exposed to intermittent stretching as a result of lung volume expansion during normal breathing. High stimulation frequency and stretching are both known to stimulate the mTOR pathway through activation of phospholipase D proteins [45]. Activation of this pathway can influence the degree of autophagy due to the inhibitory effect of mTOR on Unc-51 like kinase (Ulk1), the initiator of autophagy [46].

In summary, we report here that induction of severe sepsis in murine skeletal muscles elicits morphological and functional injuries in mitochondria that are associated with decreased biogenesis and Ca^2+^ retention capacity and increased mitochondrial recycling by the autophagy-lysosomal degradation pathway. We also report that these responses are dependent on the pattern of activation and fiber-type composition of various skeletal muscles. The DIA, which undergoes phasic activation and stretching during the breathing cycle, appears to be relatively spared, while limb muscles, which are rich in fast-twitch muscle fibers, are more severely affected.

## Supporting Information

File S1 **Supporting information describing methodologies used to measure mitochondrial H_2_O_2_ release and mitochondrial citrate synthase activity.**
(DOC)
