# Supplementary material for: Autophagy and Skeletal Muscles in Sepsis
Source: PLoS One. 2012 Oct 9;7(10):e47265. doi: 10.1371/journal.pone.0047265 (PMC3467208; doi:10.1371/journal.pone.0047265)
Supplement: File S1 — Supporting information describing methodologies used to measure mitochondrial H2O2 release and mitochondrial citrate synthase activity. (DOC) [file pone.0047265.s001.doc]

**Mitochondrial H_2_O_2_ release:**  Mitochondrial H_2_O_2_ release was determined in freshly excised diaphragm and TA muscles from 6 control C57/Bl6 mice. H_2_O_2_ release was assessed by measuring the rate of increase in Amplex-red (20 µM) fluorescence in permeabilized fibers (0.3-1.0 mg dry weight) incubated in 600 µL of K-MES buffer (in mM: 110 K-MES, 35 KCl, 1 EGTA, 5 K_2_HPO4, 3 MgCl_2_6H_2_O, and 0.5mg/ml bovine serum albumin, pH 7.3, 37 ºC) supplemented with 1.2 U/mL horseradish peroxidase. After adding the muscle fibers, the following injection sequence was performed: glutamate-malate-succinate (5 - 2.5 -5 mM), ADP (1 mM), and rotenone+antimycin-A (1 + 8 µM). Rate of H_2_O_2_ release was calculated from a standard curve established in the same experimental conditions except that fibers were absent. Results were expressed per unit of the marker enzyme citrate synthase (CS) to normalize for differences in mitochondrial content between muscle types.

**Mitochondrial citrate synthase activity assay:** Muscle samples were finely minced using small scissors and homogenized in a 200mM Tris extraction buffer containing 50 mM triethanolomine and 1 mM EDTA at pH 7.4. Citrate synthase activity was measured by detecting the increase in absorbance at 412 nm in a 96-well plate at 30°C, using 200 μl reaction buffer (200 mM Tris, pH 7.4) containing (in μm): 2 acetyl-CoA, 200 5,5′-dithiobis-(2-nitrobenzoic acid) (DTNB), 350 oxaloacetic acid and 0.1% Triton-x. The molar extinction coefficient used was 13.6 L mol^−1^ cm^−1^ for DTNB.


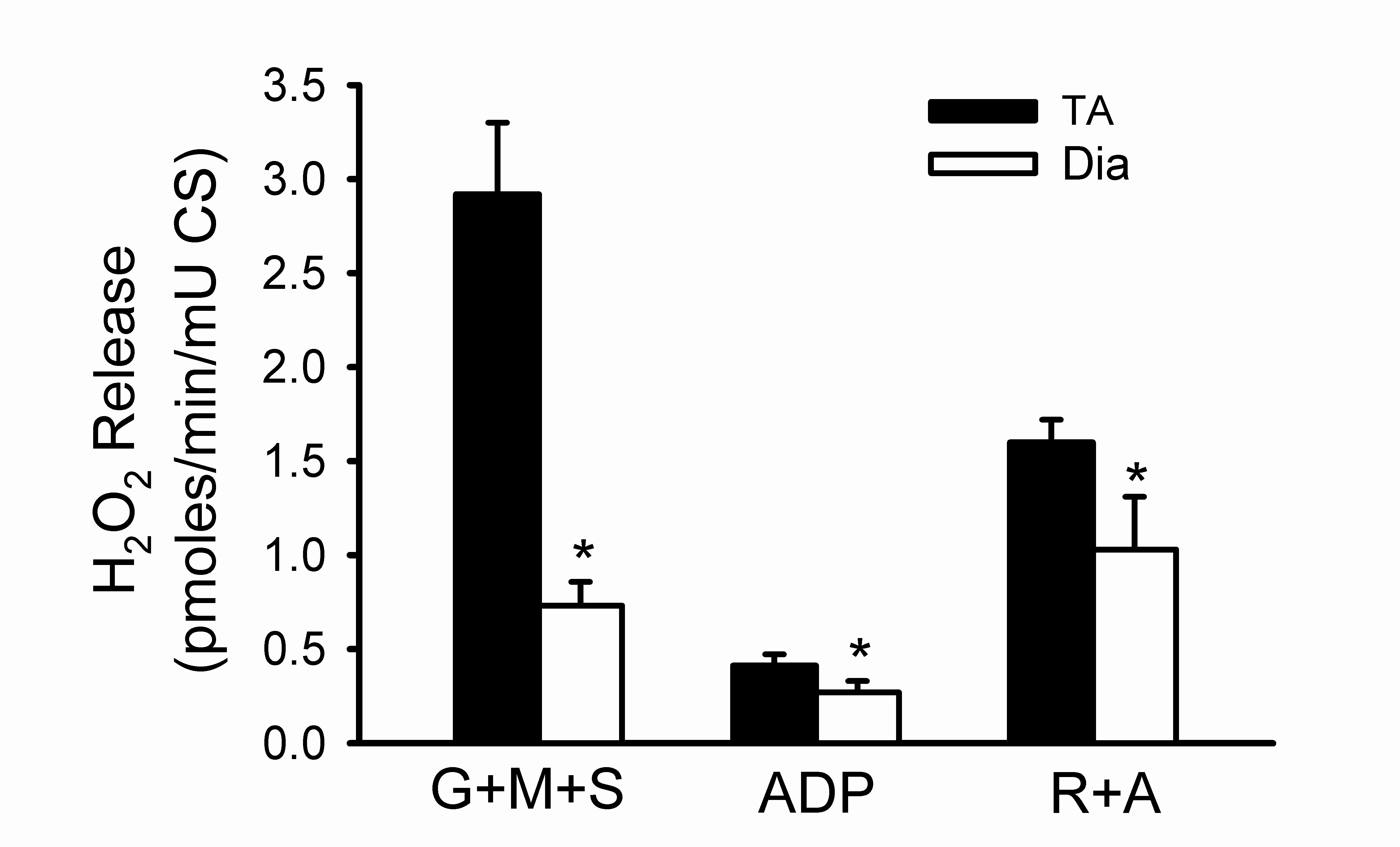


**Figure S1:** H_2_O_2_ release from muscle fibers of the diaphragm and tibialis anterior (TA) of control adult C57/Bl6 mice. *P<0.05 compared with the TA. CS: Citrate synthase; G: glutamate; M: malate; S: succinate; ADP: adenosine diphosphate; R: rotenone; and A: antimycin A.
